# *Clostridium butyricum* Prevents Diarrhea Incidence in Weaned Piglets Induced by *Escherichia coli* K88 through Rectal Bacteria–Host Metabolic Cross-Talk

**DOI:** 10.3390/ani14162287

**Published:** 2024-08-06

**Authors:** Jing Liang, Sihu Wang, Shasha Kou, Cheng Chen, Wenju Zhang, Cunxi Nie

**Affiliations:** 1 College of Life Science, Yulin University, Yulin 719000, China; lj2009133823@163.com (J.L.); sihumeng@126.com (S.W.); 2 College of Animal Science and Technology, Shihezi University, Shihezi 832003, China; 13199937005@163.com (S.K.); enze105@163.com (C.C.); 3 College of Animal Science and Technology, Northwest A&F University, Xianyang 712100, China

**Keywords:** *Clostridium butyricum*, diarrhea incidence, rectal microbiota, rectal metabolites, weaned piglet, *Escherichia coli* K88

## Abstract

**Simple Summary:**

Following weaning, piglets exhibit an immature immune system, reduced capacity to digest and absorb nutrients, and encounter diverse stressors that significantly compromise their overall health. The supplementation of probiotics in animal feed has proven to be a beneficial strategy for enhancing the welfare of livestock and poultry. The findings of this research demonstrate that supplementing the diet with 5 × 10^8^ CFU/kg of *Clostridium butyricum* enhances fecal microflora diversity and richness through the modulation of specific metabolites such as L-aspartic acid, 5-hydroxyindole-3-acetic acid, stearic acid, and adipic acid. This mechanism contributes to a reduction in the incidence of diarrhea in weaned piglets triggered by *E. coli* K88. Consequently, the study outcomes offer both empirical evidence and theoretical support for the utilization of *Clostridium butyricum* in combatting diarrhea in piglet rearing.

**Abstract:**

This study aimed to evaluate the effects of *Clostridium butyricum* (*C. butyricum*) on the prevention of the diarrhea rates and growth performances of weaned piglets induced by *Escherichia coli* K88 (*E. coli* K88). Twenty-four weaned piglets (6.92 ± 0.11 kg) were randomly assigned to one of three treatment groups for a period of 21 days. Each group consisted of eight pigs, with each pig being housed in an individual pen. Group I received the control diet along with normal saline, Group II received the control diet along with *E. coli* K88, and Group III received the control diet supplemented with 5 × 10^8^ CFU/kg of *C. butyricum* and *E. coli* K88. We examined alterations in rectal microbiota and metabolites, analyzed the incidence of diarrhea, and investigated the interactions between microbiota and metabolites through the application of Illumina MiSeq sequencing and liquid chromatography–mass spectrometry. The results showed that, from days 14 to 21, the diarrhea incidence in Group III decreased significantly by 83.29% compared to Group II (*p* < 0.05). Over the entire experimental duration, the average daily feed intake of Group III decreased significantly by 11.13% compared to Group I (*p* < 0.05), while the diarrhea incidence in Group III decreased by 71.46% compared to Group II (*p* < 0.05). The predominant microbial flora in the rectum consisted of Firmicutes (57.32%), Bacteroidetes (41.03%), and Proteobacteria (0.66%). Administering *E. coli* K88 orally can elevate the relative abundance of *Megasphaera* (*p* < 0.05). Conversely, the supplementation of *C. butyricum* in the diet reduced the relative abundance of *Megasphaera* (*p* < 0.05), while increasing the relative abundance of unclassified_f_*Lachnospiraceae* (*p* < 0.05). Rectal metabolomics analysis revealed that supplementing *C. butyricum* in the feed significantly altered the amino acids and fatty acids of the piglets infected with *E. coli* K88 (*p* < 0.05). The correlation analysis showed that the occurrence of diarrhea was inversely related to adipic acid (*p* < 0.05) and positively associated with (5-hydroxyindol-3-YL) acetic acid and L-aspartic acid (*p* < 0.05). *Prevotella*_1 exhibited a negative correlation with octadecanoic acid (*p* < 0.05). *Prevotellaceae*_UCG-005 showed a negative correlation with (5-hydroxyindol-3-YL) acetic acid (*p* < 0.05). The findings from this research study aid in probiotic development and the enhancement of healthy growth in weaned piglets.

## 1. Introduction

There are a large number of complex microorganisms in the gastrointestinal tract of animals, including bacteria, fungi, protozoa, and archaea [1]. The intestinal microbiota plays an important role in preventing the colonization of pathogens, regulating immune responses, and maintaining the stability of the gastrointestinal environment [1]. The structure and composition of the gut microbiota are influenced by factors such as genetics, diet, age, external environment, and stress [2]. Newborn piglets are prone to diarrhea in the early stages of gastrointestinal development [3]. Before birth, the fetal intestine is thought to be sterile, but newborn infants are colonized by a complex microbial community shortly after birth [4]. Imbalances or dysbiosis in the neonatal gut microbiota can lead to a higher risk of disease and have adverse effects on host health [5]. Increasing evidence suggests that an early colonization of the microbiota can affect its composition and immune maturation [6,7]. Therefore, early colonization with beneficial bacteria to establish a stable gut microbiota may have practical significance in improving the health of newborn piglets. Newborn piglets are often infected with pathogenic bacteria from maternal or environmental sources [8]. Enterotoxigenic *Escherichia coli* (ETEC) K88 is considered a major pathogen for post-weaning diarrhea in piglets [9]. ETEC K88 infection is one of the key causes of diarrhea in both newborn and post-weaning piglets [3], leading to slow growth in pig farms and causing significant economic losses [10]. The severity of diarrhea in suckling piglets can be as high as 50% [11]. ETEC produces enterotoxins, disrupts the gut microbiota, stimulates the loss of water and electrolytes, and causes diarrhea [12]. Additionally, when weaned piglets experience intestinal damage, there may be changes in the regulation of their metabolism [13].

Probiotics can promote the proliferation of anaerobic bacteria and inhibit or eliminate harmful bacteria through competing for nutrients and growth sites, thereby continuously reducing the number of harmful bacteria and rendering them incapable of causing disease in the body [14]. *Clostridium butyricum* (*C. butyricum*) is an important component of probiotics, mainly residing in the distal small intestine and colon of animals [15], and has been used to prevent or treat gastrointestinal diseases in animals [16]. Studies have shown that adding *C. butyricum* to feed can regulate the gut microbiota of broiler chickens, increasing the numbers of certain beneficial bacteria and reducing the numbers of harmful bacteria in the chicken gut [17,18]. Furthermore, the liver-protective effect of *C. butyricum* in mice is believed to be related to its reshaping of the gut microbiota composition [19]. In recent years, metabolomics has been introduced into the study of the nutritional metabolism of post-weaning piglets [20] and finishing pigs [21]. This study advances a scientific hypothesis suggesting that *C. butyricum* is capable of colonizing the intestinal tract and inhibiting the growth of detrimental bacteria, thereby preserving the intestinal microecological balance and enhancing the healthy growth of piglets. Building on the previous research of our research group, this investigation aims to establish an *E. coli* K88 diarrhea model to explore the diversity and changes in the fecal microbiota of diarrhea piglets mediated by *E. coli* K88, the co-variation characteristics of the microbiota and host metabolism, and to identify important fecal bacterial types and biomarkers closely associated with host diarrhea.

## 2. Materials and Methods

### 2.1. Animals, Diet, and Management

A total of 24 weaning piglets [(Yorkshire × Landrace) × Duroc] (half male, half female) were weaned at 28 days of age (6.92 ± 0.11 kg BW) and randomly divided into three groups for 21 days, with eight pigs per group. Each pig was fed in a single pen, receiving a control diet (Group I), a control diet (Group II), or a diet supplemented with 5 × 10^8^ CFU/kg *C. butyricum* (Group III). On the 18th day of the experiment, piglets in Group I were gavaged (force-fed) sterile saline, and the other two groups’ piglets were gavaged *E. coli* K88 for three consecutive days. The diets were a highly digestible basal diet, including highly digestible carbohydrate ingredients and low, anti-nutritional protein ingredients. The composition and nutrient content of the treatment diets are presented in Table 1. The *C. butyricum* culture was provided by Zhejiang Huijia Biological Technology Co., Ltd. (Anji, Zhejiang, China). The commercial product contained a viable count of 5.0 × 10^8^ CFU/g.

The pigs were housed in a climate-controlled facility with regulated temperature and humidity to minimize environmental stress. The room temperature was maintained at 28–30 °C for the first week and then gradually reduced to 22–24 °C. Humidity was kept between 55 and 65%. All pigs were subjected to a 12 h light/dark cycle and had ad libitum access to water and their respective diets. Regular cleaning and disinfection protocols were strictly followed to maintain hygiene and reduce the risk of disease. This experiment was carried out at Fushun Breeding Farmer Professional Cooperative, a pig farm located in Shihezi, Xinjiang.

### 2.2. Oral Challenge

The *E. coli* K88 strain was obtained from the Jiangsu Academy of Agricultural Sciences (Nanjing, China) and grown at 37 °C. The piglets fasted for 12 h before modeling. Firstly, 10 mL of 1% sodium bicarbonate solution was gavaged, and, after one hour, *E. coli* K88 10 mL (1.0 × 10^10^ CFU/mL) was gavaged once a day for three consecutive days. Group I was treated with sterile saline in the same way.

### 2.3. Sample Collection

Body weight per pen was recorded on days 1, 7, 14, and 21 to calculate average daily gain (ADG). Feed was weighed and provided daily, and the feed consumption was determined on day 7, day 14, and day 21 by weighing the remaining feed in the feeder. The average daily feed intake (ADFI) and feed-to-gain ratio (F/G) of each phase were calculated. In the experimental period, the piglets were observed daily for the presence of diarrhea: the number of piglets experiencing diarrhea per day in each pen was recorded, and the consistency of the feces was simultaneously evaluated according to 4 levels: 0, normal; 1, pasty; 2, semi-liquid; and 3, liquid [22]. Diarrhea was defined as fecal output at level 2 or 3 for two consecutive days. Fecal consistency was visually assessed daily. Diarrhea incidence of each group (%) = the total number of days of diarrhea of piglets in the group during the experiment/(the number of piglets in the group × the number of days of the experiment) × 100%

On day 21 of the experiment, six piglets in each group were randomly selected. About 20 g of fresh feces was collected by rectal stimulation method and stored in sterile cryopreservation tubes, which were immediately placed in liquid nitrogen, and stored at −80 °C in a refrigerator for further analysis. Six samples were employed for the analysis of metabolites, and, from these, three samples were randomly chosen for 16S rRNA high-throughput sequencing.

### 2.4. DNA Extraction

Using a TIANamp Stool QIAamp PowerFecal DNA Kit (QIAGEN, Hilden, Germany), microbial DNA was extracted from fecal samples according to the instructions in the kit. NanoDrop 2000 UV-VIS spectrophotometer (Thermo Scientific, Wilmington, NC, USA) DNA concentration and purity were determined, and DNA quality and concentration were determined by 1% agarose gel electrophoresis.

### 2.5. Illumina MiSeq Sequencing

Using a PCR thermocycling system (GeneAmp 9700, ABI, Los Angeles, CA, USA), the samples of bacterial 16S rRNA hypervariable region were amplified with primers 515F (5′-GTGCCAGCMGCCGCGGTAA-3′) and 907R (5′-CCGTCAATTCCTTTGAGTTT-3′) and sent to Shanghai Magi Biomedical Technology Co., Ltd., Shanghai, China. Illumina sequencing was performed on the MiSeq platform (Illumina, San Diego, CA, USA). The raw sequencing data were deposited in the NCBI Sequence Read Archive (SRA, http://www.ncbi.nlm.nih.gov/sra) under the accession number PRJNA679230 accessed on 18 November 2020.

### 2.6. Data Processing

Raw FASTQ files were quality-filtered by Trimmomatic software (Version 0.36) and combined by Quick Length Adjustment (FLASH) for short reads according to the following criteria. UPARSE software (Version 7.1, http://drive5.com/uparse/, accessed on 18 November 2020), clustering, was used according to the similarity of the 97% cutoff value for OTU. The 16S rRNA gene sequences were classified and analyzed using the Ribosomal Database Project (RDP) classification algorithm (http://rdp.cme.msu.edu/, accessed on 18 November 2020) and SILVA 16S rRNA gene reference database (https://www.arb-silva.de/, accessed on 18 November 2020), with a confidence threshold of 70%.

### 2.7. Extraction of Metabolites

First, 100 mg of feces was placed in a 5 mL tube, and 500 μL of dd H_2_O was added at 4 °C and thoroughly mixed for 1 min. In the second step, 1 mL of methanol at −20 °C was added to vortex for 30 s. The third step is to place the ultrasonic machine at room temperature for 10 min and then place it on ice for 30 min. The fourth step was to centrifuge at 14,000 rpm at 4 °C for 10 min; 1200 μL of supernatant was taken and transferred to a new 1.5 mL centrifuge tube. The sample was concentrated with a vacuum centrifuge concentrator. In the fifth step, the sample was dissolved in 400 μL of methanol solution at 4 °C in a ratio of 1:1 and filtered with a 0.22 μm membrane to obtain the sample for testing. Then, transfer 20 μL from each sample to be tested and mix it into the QC sample (QC: quality control, used to correct the deviation of the analysis’ result of the mixed sample and the error caused by the analytical instrument itself). Finally, the remaining samples were tested using LC–MS.

### 2.8. Chromatographic Condition

The Waters ACQUITY UPLC (ACQUITY UPLC)^®^ HSS T3 1.8 μm (2.1 × 150 mm) column was utilized for analysis. The automatic sampler temperature was maintained at 4 °C, with a flow rate set to 0.25 mL/min, while the column temperature was kept at 40 °C during the 40 μL gradient elution. The mobile phase consisted of 0.1% formic acid in water (A) and 0.1% formic acid in acetonitrile (B). The gradient elution profile included the following steps: 0–1 min, 2% B; 1–9.5 min, 2–0% B; 9.5–14 min, 50–98% B; 14–15 min, 98% B; 15–15.5 min, 98–2% B; 15.5–17 min, 2%.

### 2.9. Mass Spectrometry Conditions

Thermo LTQ Orbitrap XL, electrospray ion source (ESI), positive and negative ionization mode, positive ion spray voltage 4.80 kV, negative ion spray voltage 4.50 kV, sheath gas at 45 arb, and auxiliary gas at 15 arb were applied. The capillary temperature was 325 °C, the capillary voltage was 35 V/−15 V, and the tube lens voltage was 50 V/−50 V. The full scan was carried out with a resolution of 60,000, and the scanning range was 50~1000. Secondary cracking was carried out with CID, and the collision voltage was 30 eV. At the same time, the unnecessary MS/MS information was removed by dynamic exclusion (the repeat count was 2), and the dynamic exclusion time was set to 15 s.

### 2.10. Data Processing

ProteoWizard software (Version 3.0.8789) was used to change the original file to MzXML format (XCMS input file format). R’s XCMS package (Version 3.3.2) was used for peak detection peak filtering and peak alignment. Then, a two-dimensional data matrix consisting of the mass/charge ratio (*m*/*z*) residence time (RT) and peak area of metabolites were obtained. SIMCA-P 13.0 software (Umetrics AB, Umea, Sweden) was introduced for multivariate statistical analysis. Partial least-squares discriminant analysis (PLS-DA) was used for predictive description modeling and discriminant variable selection. The significant variables under differential multiples (FC ≥ 1.5 or FC ≤ 0.667) obtained by OPLS-DA and *t* test (*p* < 0.05) were used to analyze the metabolites differentially expressed in the two groups. Then, accurate MS/MS fragment information was searched for in the human metabolome database (HMDB, http://www.hmdb.ca/, accessed on 18 November 2020), Metlin database (http://metlin.scripps.edu/, accessed on 18 November 2020), and KEGG database (http://www.genome.jp/kegg/, accessed on 18 November 2020) for screening and identification of potential biomarkers. Metabolic pathways were analyzed by MetaboAnalyst R 3.0, available at http://www.metaboanalyst.ca, accessed on 18 November 2020.

### 2.11. Bioinformatics and Statistical Analyses

The growth performance data and alpha diversity index data were reported as mean ± standard deviation (SD), with one-way analysis of variance (ANOVA) with Duncan’s multiple range test. The diarrhea rate data were analyzed using the chi-square test. The rank–abundance and rarefaction curves were made by using R language tools based on Majorbio Cloud (https://cloud.majorbio.com/, accessed on 18 November 2020). The software Mothur (version v.1.30.2; https://mothur.org/wiki/calculators/, accessed on 18 November 2020) was used to perform α-diversity index analysis. Community composition analysis involved plotting with R language tools (version 3.3.1). Heat maps were generated using the VEGAN R package (Version 3.3.1), and the VEGAN package facilitated non-metric multidimensional scaling (NMDS) analysis. Graphical representations, like histograms and Venn diagrams, were created through Origin 8.0 software by OriginLab. Spearman’s Rho test assessed the relationship between bacteria displaying significant changes and their metabolites (with FC exceeding 0.667 or 1.5, after adjustment, and *p* < 0.05). The correlation map illustrating the link between microbiota and metabolites was produced using the Jidiao cloud platform available at https://www.omicshare.com, accessed on 18 November 2020. The statistical analysis for this experiment utilized SPSS version 22.0. A significance level of *p* ≤ 0.01 indicated a highly significant difference, *p* ≤ 0.05 indicated a significant difference, and *p* > 0.05 signified an insignificant difference.

## 3. Results

### 3.1. Growth Performance and Diarrhea Incidence

At 0–7 and 7–14 days post-weaning, there were no significant differences in feed conversion efficiency and diarrhea incidence among all experimental groups (*p* > 0.05). However, from 14 to 21 days after weaning, the average daily feed intake (ADFI) of group III decreased significantly by 17.47% compared to group II (*p* < 0.05), while the occurrence of diarrhea in group III decreased significantly by 83.29% compared to group II (*p* < 0.05). Over the entire duration of the experiment, the ADFI in group III decreased significantly by 11.13% compared to group I (*p* < 0.05), and the incidence of diarrhea in group III decreased significantly by 71.46% compared to group II (Table 2).

### 3.2. The Fecal Microbial Community Structure in Weaned Piglets Challenged with E. coli K88

**Figure 1:**
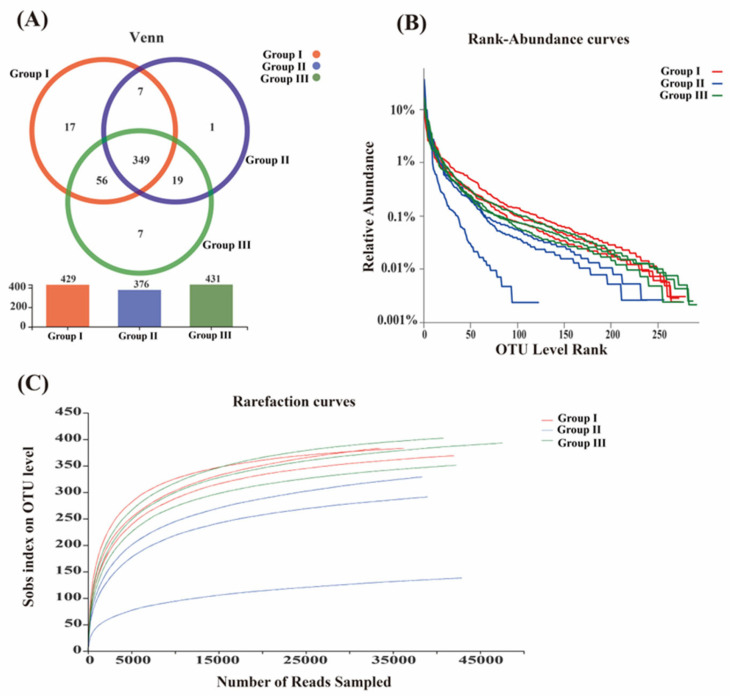
Venn diagram, rank–abundance and rarefaction curves of fecal microorganism in weaned piglets in different treatment groups. (**A**) Venn diagram; (**B**) rank–abundance curve; (**C**) rarefaction curve.

Based on a 97% similarity threshold, all sequences were clustered into operational taxonomic units (OTUs), resulting in the identification of a total of 456 OTUs. Notably, 349 OTUs were shared among the three treatment groups, representing 76.54% of the total OTUs, while 82 OTUs were common between the two treatment groups, accounting for 17.98% of the total OTUs (Figure 1).

The sequencing coverage rate for each treatment group exceeded 99%, suggesting a high probability of sequence detection in the samples and a low likelihood of missed detection. These sequencing results accurately reflect the microbial composition of the samples. The Sobs index in group I and group III increased significantly by 22.04% and 23.23%, respectively, compared to group II (*p* < 0.05). Furthermore, the Shannon index in group I showed a significant increase of 20.35% compared to group II (*p* < 0.05). No significant differences were observed in the Ace and Chao indices among the various treatments (*p* > 0.05), indicating that the *C. butyricum* (CB) treatment group displayed high richness and diversity (Table 3).

**Figure 2:**
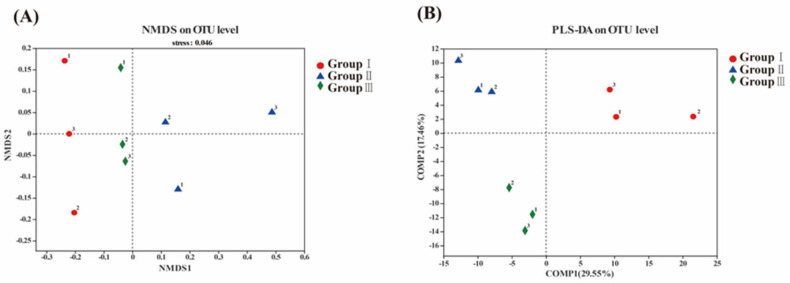
Non-metric multidimensional scaling analysis (**A**) and partial least-squares discriminant analysis (**B**) at the OTU level for three treatment groups.

In Figure 2A, each sample from the three groups is distributed and clustered at the operational taxonomic unit (OTU) level with relatively minor discrepancies. The partial least-squares discriminant analysis (PLS-DA) effectively distinguishes the observed values between groups and identifies the influential variables contributing to between-group differences. The distinct separation of the three treatment groups without sample overlap is evident in Figure 2B. The suspected influencing factor for microbial compositional divergence between group I and group II was Comp1, with the explanatory weight ratio of each dimension on the outcomes. Additionally, the suspected influential factor associated with group II and group III was *C. butyricum*, denoted as Comp2. The combination of the sample information revealed ETEC K88 as a potential influential factor for group I (Figure 2).

**Figure 3:**
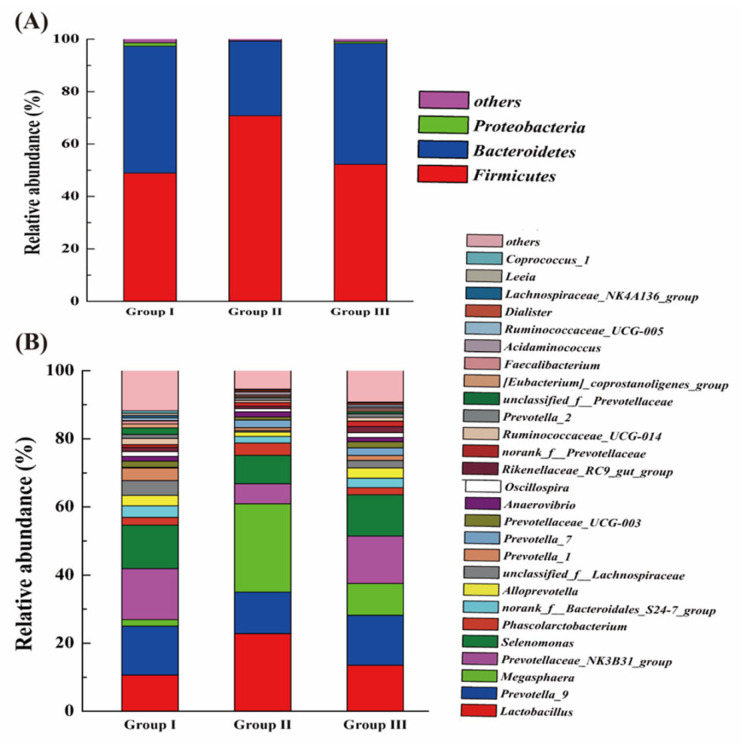
Classification of the bacterial community composition across the three different treatment groups. (**A**) Relative abundance of bacterial phylum level and (**B**) relative abundance of bacterial genus level.

Figure 3A displays three categories of microorganisms—Firmicutes, Bacteroidetes, and Proteobacteria—with average abundances of 57.32%, 41.03%, and 0.66%, respectively. At the genus level (Figure 3B), 141 genera were identified, including *Lactobacillus* (15.68%), *Prevotella*_9 (13.72%), and *Megasphaera* (12.38%). The relative abundance of unclassified_f_*Lachnospiraceae* in group III significantly exceeded that in group II (*p* < 0.001), while the relative abundance of *Megasphaera* in group III notably decreased compared to group II (*p* = 0.047) (Figure 3).

**Figure 4:**
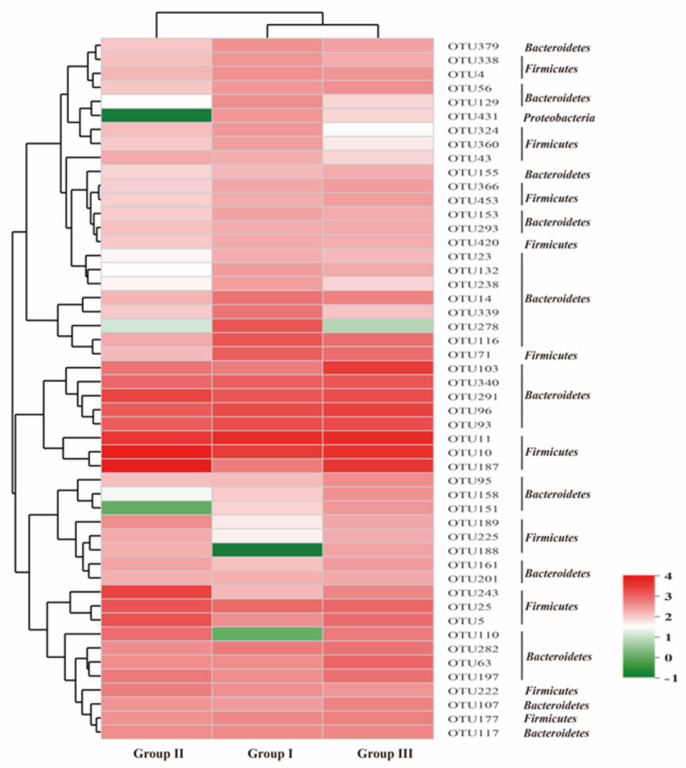
Heatmap showing the most relative abundance of dominant bacterial OTUs. Note: the relative values are indicated by color intensity, with the legend indicated at the right corner.

Figure 4 illustrates the bacterial communities of the three treatment groups, grouping them distinctly: group II forms one cluster, while group I and group III constitute another, signifying the high similarity and separate clustering of the bacterial communities in group I and group III from group II (Figure 4).

### 3.3. Fecal Metabolites

**Figure 5:**
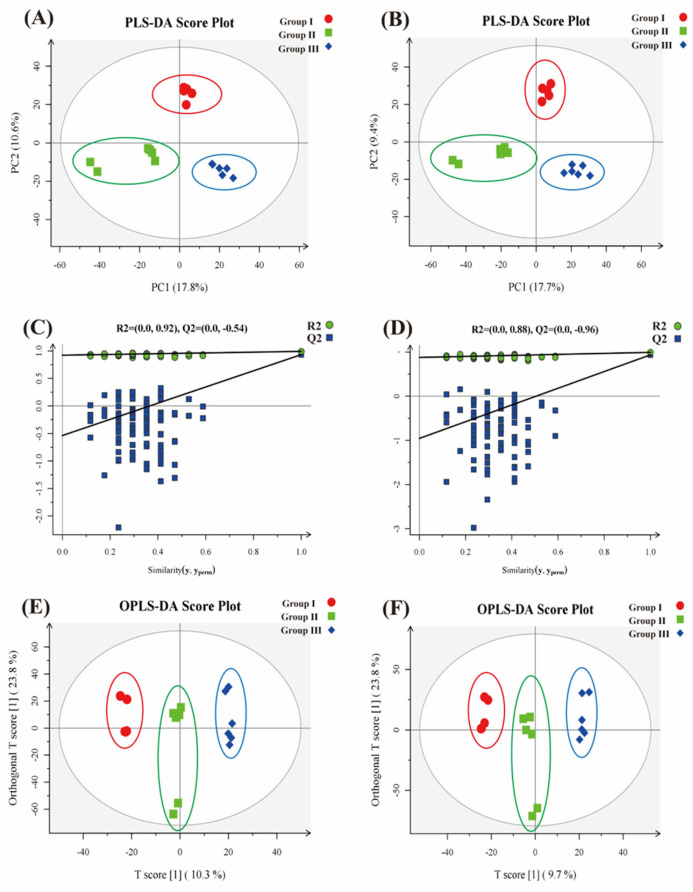
Two-dimensional score plots of fecal metabolites in three different treatment groups. (**A**,**B**) partial least-squares discriminant analysis (PLS-DA) in positive/negative ion modes, (**C**,**D**) PLS-DA replacement test in positive/negative ion mode, and (**E**,**F**) orthogonal partial least-squares discriminant analysis (OPLS-DA) in positive/negative ion mode (*n* = 6).

Figure 5A,B present PLS-DA models for dimension reduction analysis. In each figure, every point represents a sample. The PLS-DA model yields the following results: R2X (cum) = 0.531, R2Y (cum) = 0.992, and Q2 (cum) = 0.933 in positive ion mode; and R2X (cum) = 0.563, R2Y (cum) = 0.993, and Q2 (cum) = 0.937 in negative ion mode. The Permutations Plot aids in effectively assessing the potential overfitting of the current PLS-DA model. An essential criterion is that all blue Q2 values should be lower from left to right compared to the original blue Q2, or the regression line of Q2 should be at or below zero at the vertical coordinate intersection. Figure 5C (positive ion mode) and Figure 5D (negative ion mode) support the reliability and effectiveness of this model. An additional OPLS-DA analysis was conducted to further diminish intra-group differences and accentuate inter-group distinctions. Illustrated in Figure 5E,F, the model yields the following parameters: R2X (cum) = 0.415, R2Y (cum) = 0.996, and Q2 (cum) = 0.831 in positive ion mode; and R2X (cum) = 0.335, R2Y (cum) = 0.991, and Q2 (cum) = 0.803 in negative ion mode. The fecal metabolite score maps, constructed within the 95% Hotelling T2 confidence ellipse, display clear separation, indicating a strong identification and prediction accuracy of the model. The scatter plot division among the three treatment groups indicates significant differences in the composition and concentration of the variables or molecules present in the samples (Figure 5).

Table 4 illustrates two distinct metabolites between group I and group II: L-aspartic acid and (5-hydroxyindol-3-YL) acetic acid. Group I and group III exhibited four metabolites that varied, identified as L-phenylalanyl-L-proline, 2-keto-3-deoxy-D-gluconic acid, octadecanoic acid, and CMP. The comparison between group II and group III revealed four differential metabolites, specifically L-aspartic acid, (5-hydroxyindol-3-YL) acetic acid, octadecanoic acid, and adipic acid. Significantly higher levels of L-aspartic acid and (5-hydroxyindol-3-YL) acetic acid were observed in group II compared to group III (*p* < 0.05), while octadecanoic acid and adipic acid levels notably decreased (*p* < 0.05).

**Figure 6:**
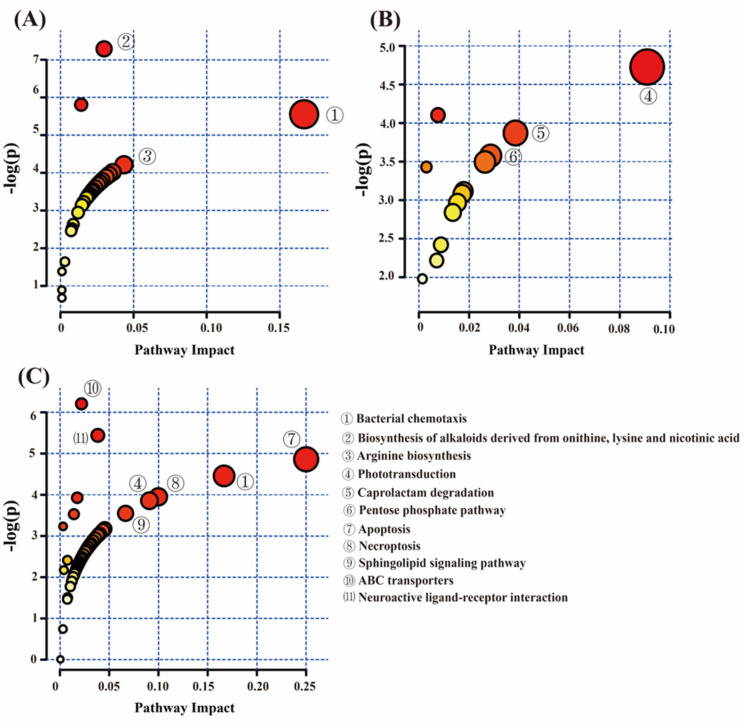
Pathway enrichment map analysis of differential metabolites in feces between (**A**–**C**) using MetaboAnalystR 3.0. Note: the color of the circles from white to yellow to red denotes incremental fold change (−log(*p*)). The size of the circles from small to large indicates an increment of the impact of the pathway.

MetaboAnalystR 3.0 was utilized for the pathway enrichment profiling of the distinct metabolites. The metabolites in group I and group II were primarily associated with bacterial chemotaxis, biosynthesis of alkaloids for tannin production, lysine, nicotinic acid, and arginine biosynthesis pathways (Figure 6A). Differential metabolites within group I and group III were predominantly linked to photoconduction, caprolactam degradation, and pentose phosphate pathway metabolism (Figure 6B). In contrast, the differential metabolites between group II and group III were primarily involved in metabolic pathways such as apoptosis, necrotic apoptosis, sphingolipid signaling, bacterial chemotaxis, ABC transporters, and interactions in neural tissues (Figure 6C).

### 3.4. Analysis of the Correlation between Diarrhea Incidence, Body Weight, Fecal Microbial Community, and Fecal Metabolites

**Figure 7:**
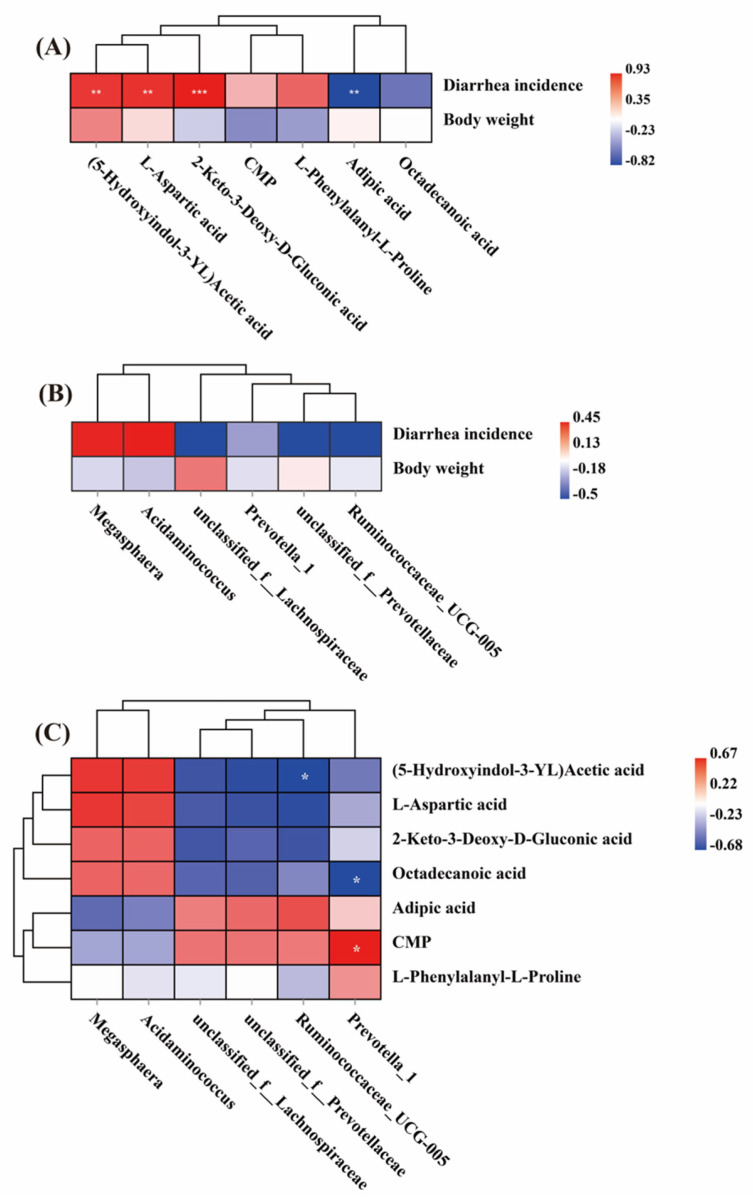
Correlation between the body weight, diarrhea incidence, differential microbiota (at the genera level), and metabolites. (**A**) Correlation between body weight, diarrhea incidence, and microbiota. (**B**) Correlation between body weight, diarrhea incidence, and metabolites. (**C**) Correlation between microbiota and metabolites. Note: the strength (Spearman’s ρ value) and significance of correlations are shown as color in shades (red, positive correlation; blue, negative correlation). The values above/below zero represent positive/negative correlations. Significant correlations are noted by * *p* ≤ 0.05, ** *p* ≤ 0.01, *** *p* ≤ 0.001.

The Spearman correlation analysis results are depicted in Figure 7. The diarrhea rate exhibited a negative correlation with adipic acid (*p* < 0.05) and was positively correlated with 5-hydroxyindole-3-acetic acid, 2-keto-3-deoxy-D-gluconic acid, and L-aspartic acid (*p* < 0.05) (Figure 7B). Furthermore, Prevotella demonstrated a positive correlation with cytidine acid (*p* < 0.05) and a negative correlation with stearic acid (*p* < 0.05). Additionally, Prevotellaceae showed a negative correlation with 5-hydroxyindole-3-acetic acid (*p* < 0.05) (Figure 7C).

## 4. Discussion

### 4.1. The Impact of C. butyricum on the Growth Performance and Diarrhea Rate Induced by Escherichia coli K88 in Weaned Piglets

Post-weaning diarrhea presents a significant threat to piglet production, associated with the intestinal colonization of enterotoxigenic *Escherichia coli* (ETEC), leading to gut dysbiosis, inflammation, growth retardation, and potential mortality [23]. ETEC poses a crucial challenge in the swine industry, impacting post-weaning performance and piglet survival rates [24], possibly through immune-barrier enhancement and gut microbiota alterations. Probiotics and organic acids can regulate cytokine production by inhibiting ETEC K88 proliferation and toxin release, thereby ameliorating the inflammatory response [25,26]. Research suggests that supplementing weaned piglet diets with *C. butyricum* has been shown to enhance growth performance in pigs fed less digestible diets. There was a noticeable trend towards a reduction in the feed-to-gain ratio (F/G), which could potentially lead to cost savings in pig production. The positive effects may be attributed to a decrease in post-weaning diarrhea, which is associated with improvements in intestinal morphology, intestinal microflora composition, and immune function [27]. A study by Han [28] demonstrated that supplementing feed with *C. butyricum* enhances growth performance in weaned piglets, improves feed efficiency, reduces the feed-to-gain ratio significantly, and decreases diarrhea incidence. Numerous studies have demonstrated that *C. butyricum* can enhance growth performance and optimize nutrient utilization in animals [29,30,31]. However, some research findings have not observed an impact on growth performance [32,33,34]. In this study, the incorporation of *C. butyricum* in feed had no significant impact on feed efficiency but notably reduced the diarrhea rate in piglets, likely attributed to the production of volatile fatty acids like butyric acid, which lower intestinal pH and impede the growth of harmful bacteria.

### 4.2. The Impact of C. butyricum on the Fecal Microbiota of Weaned Piglets Stimulated by Escherichia coli K88

The equilibrium of the animal intestinal microbiota is crucial for sustaining overall animal health. This delicate balance is vulnerable to interference from external pathogens. Should this balance be disrupted, it can result in the onset of various diseases within the body. Adding probiotics to the diet can enhance the intestinal microbiota [35,36], improve gastrointestinal function [37], and boost systemic immunity [38], consequently enhancing animal production performance [37]. Studies have demonstrated that prolonged antibiotic use can modify the structure and composition of the gut microbiota, leading to a decreased expression of immune response genes [9,39]. With the implementation of widespread antibiotic restrictions, probiotics, prebiotics, synbiotics, and other supplements serve as antibiotic alternatives, proven to regulate the intestinal microbiota and enhance the well-being of piglets. *C. butyricum* is adept at maintaining this microecological equilibrium by enhancing the population of beneficial bacteria and impeding the colonization and growth of pathogens. It also works in concert with other beneficial bacteria to neutralize harmful bacteria, vie with pathogens for colonization sites, and diminish the probability of pathogen colonization, thereby checking the growth of pathogens. The metabolic activities of *C. butyricum* yield compounds such as short-chain fatty acids (SCFAs) and antimicrobial peptides that exert inhibitory effects on pathogens. SCFAs can reduce the pH level of the intestinal environment, which in turn suppresses the proliferation of pathogens. Additionally, bacteriocins and antimicrobial peptides can weaken the colonization potential of pathogens or directly assault them, thereby safeguarding intestinal health [40,41].

In the intestinal microbiota of weaned piglets, the main phyla are Firmicutes and Bacteroidetes [42,43]. The majority of Bacteroidetes can decompose starch, hemicellulose, and protein. They hydrolyze indigestible dietary polysaccharides into smaller molecules that are more easily absorbed by the body, providing supplementary energy [44]. Hossain [45] administered a composite probiotic blend of *C. butyricum*, *Bacillus subtilis*, and *Lactobacillus* to broilers. The study revealed that probiotic supplementation significantly increased *Lactobacillus* and *Bifidobacterium* counts, while notably inhibiting the growth of *Escherichia coli* and *Clostridium* perfringens. Here, the higher 0.2% dosage exhibited superior inhibitory effects compared to the lower 0.1% dose, outperforming the antibiotic group. The dietary supplementation with *C. butyricum* or *Enterococcus faecalis* appears to have conferred several advantages to the weaned piglets challenged with lipopolysaccharide (LPS). The supplements enhanced the pigs’ growth performance, bolstered their immune response, alleviated damage to the intestinal villi, reduced inflammation, and improved the balance of the intestinal microbiota [46].

The *Lachnospiraceae* family is a fundamental component of the gut microbiota, colonizing the intestinal lumen from birth and expanding in terms of species diversity and relative abundance throughout the host’s life span [47]. *Lachnospiraceae* can produce large amounts of SCFAs, such as acetic acid by metabolizing L-glutamic acid [48]. *Megasphaera* is a primary lactic acid-utilizing bacterium. It can decompose lactic acid into propionate via two pathways: acrylic acid and succinate. Alternatively, it can produce acetic acid through the acetyl–CoA pathway with the action of acetyl kinase (AK) and phosphotransacetylase (PTA). Furthermore, acetic and propionic acids are converted into butyric and valeric acids [49,50]. In this investigation, the addition of 5 × 10^8^ CFU/kg *C. butyricum* in the diet of diarrheic piglets markedly enhanced the diversity of the fecal microbiota, substantially raised the relative abundance of unclassified_f_*Lachnospiraceae*, and significantly decreased the relative abundance of *Megasphaera*. This may be due to the ability of *C. butyricum* to improve the stability of the animal’s digestion environment, thereby enhancing the capacity to recover health from a state of diarrhea.

### 4.3. The Influence of C. butyricum on Fecal Metabolites in Weaned Piglets Affected by Escherichia coli K88

The metabolome serves as a comprehensive reflection of the genome, transcriptome, and proteome and is instrumental in exploring the metabolic pathways within biological systems. Aspartic acid, an α-amino acid, acts as a precursor for synthesizing amino acids like lysine, threonine, isoleucine, and methionine, as well as purine and pyrimidine bases. Additionally, it can function as a crucial excitatory neurotransmitter receptor in the central nervous system of mammals [51]. Stearic acid, a naturally abundant fatty acid, is found in various fats, particularly in animal fats. Adipic acid, a valuable dicarboxylic acid within the fatty acid group, holds wide-ranging applications in medicine, pesticides, and fuels [52]. The results of fecal metabolite analysis from this study reveal that the supplementation of *C. butyricum* to the diet of diarrheic piglets leads to the presence of four distinct fecal metabolites, namely, L-aspartic acid, 5-hydroxyindole-3-acetic acid, stearic acid, and adipic acid, which serve as potential biomarkers. The major metabolic pathways involved encompass cell apoptosis, necrotic apoptosis, sphingolipid signaling pathways, and bacterial chemotaxis. The correlation analysis demonstrates a negative association between diarrhea incidence and adipic acid levels, as well as a positive relationship with L-aspartic acid and 5-hydroxyindole-3-acetic acid. This suggests that *C. butyricum* in weaned piglets may offer protection against *E. coli* K88 infection by decreasing diarrhea occurrence through elevating adipic acid levels and reducing L-aspartic acid and 5-hydroxyindole-3-acetic acid levels.

## 5. Conclusions

The supplementation of the diet with 5 × 10^8^ CFU/kg of *C. butyricum* significantly decreases the occurrence of diarrhea induced by *Escherichia coli* K88 in weaned piglets. This supplementation also enhances fecal microbiota diversity and richness in piglets affected by *E. coli* K88. This is mainly related to the metabolism of L-aspartic acid, 5-hydroxyindole-3-acetic acid, stearic acid, and adipic acid. *C. butyricum* could potentially serve as a feasible alternative to antibiotics for preventing *E. coli* K88 infection. The outcomes of this study provide empirical evidence and a theoretical basis for the application of *C. butyricum* in preventing diarrhea in piglet production.

## Figures and Tables

**Table 1:** Composition and nutrient levels of control diet (air-dried basis, %).

Ingredients	Content	Nutrient Levels ^2^	Content
Corn	58.60	Digestible energy (MJ/kg)	13.68
Soybean meal	17.50		
Expanded soybean	7.50	Crude protein	20.40
Milk powder	4.00	Lys	1.30
Fish meal	4.00	Met + Cys	0.78
Whey powder	4.30	Thr	0.87
NaCl	0.30	Na	0.27
Limestone	1.22	Ca	0.95
CaHPO_4_	1.16	Available P	0.39
DL-met	0.07		
Lys HCL	0.35		
Premix ^1^	1.00		
Total	100.00		

^1^ The premix provided the per kg of diets as follows: VA 8 000 IU, VB_1_ 4 mg, VB_2_ 3.6 mg, VB_5_ 40 mg, VB_6_ 4 mg, VB_12_ 0.02 mg, VD_3_ 3 000 IU, VE 20 IU, VK_3_ 2 mg, biotin 0.15, folic acid 1 mg, D-pantothenic acid 11 mg, nicotinic acid 10 mg, antioxidant 100 mg, Cu (as copper sulfate) 10 mg, Fe (as ferrous sulfate) 80 mg, Mn (as manganese sulfate) 30 mg, Zn (as zinc sulfate) 75 mg, I (as potassium iodide) 0.4 mg, and Se (as sodium selenite) 0.3 mg. ^2^ Digestible energy and Available P were calculated values, while the others were measured values.

**Table 2:** Effects of *C. butyricum* supplementation on growth performance and diarrhea of weaned piglets challenged with *Escherichia coli* K88.

	Treatments ^2^			
Item ^1^	Group I	Group II	Group III	*p*-Value
Initial weight (kg)	6.91 ± 0.62	6.90 ± 0.28	6.96 ± 0.59	0.968
Final weight (kg)	14.65 ± 2.28	14.11 ± 1.25	13.94 ± 1.75	0.735
d 0–7				
ADG (g)	349.11 ± 34.03	347.96 ± 46.60	366.33 ± 53.57	0.696
ADFI (g)	505.88 ± 39.58	461.86 ± 66.17	477.43 ± 21.42	0.194
F/G	1.46 ± 0.14	1.33 ± 0.12	1.33 ± 0.19	0.185
Diarrhea incidence (%)	1.79	1.79	1.79	1.000
d 7–14				
ADG (g)	386.61 ± 60.66 ^a^	321.43 ± 43.45 ^b^	337.76 ± 34.43 ^ab^	0.042
ADFI (g)	497.38 ± 30.97 ^a^	420.14 ± 47.57 ^b^	439.14 ± 58.22 ^b^	0.011
F/G	1.32 ± 0.22	1.32 ± 0.19	1.31 ± 0.19	0.992
Diarrhea incidence (%)	0	0	0	-
d 14–21				
ADG (g)	370.54 ± 86.09	360.20 ± 63.33	291.84 ± 59.25	0.101
ADFI (g)	584.63 ± 94.54 ^ab^	599.29 ± 83.80 ^a^	494.57 ± 93.23 ^b^	0.088
F/G	1.60 ± 0.14	1.68 ± 0.13	1.70 ± 0.09	0.312
Diarrhea incidence (%)	3.57 ^ab^	10.71 ^a^	1.79 ^b^	0.065
d 0–21				
ADG (g)	368.75 ± 41.58	343.20 ± 21.57	331.97 ± 32.38	0.116
ADFI (g)	529.29 ± 45.32 ^a^	493.76 ± 37.58 ^ab^	470.38 ± 31.56 ^b^	0.027
F/G	1.46 ± 0.11	1.44 ± 0.10	1.45 ± 0.12	0.955
Diarrhea incidence (%)	1.79 ^ab^	4.17 ^a^	1.19 ^b^	0.075

^1^ ADG, average daily gain; ADFI, average daily feed intake; F/G, feed-to-gain ratio. ^2^ Piglets were fed different diets. Group I, piglets fed the control diet + normal saline; group II, piglets fed the control diet + *Escherichia coli* K88; group III, piglets fed the control diet supplemented with *C. butyricum* + *Escherichia coli* K88. ^a, b^ Means in the same row with different superscript letters differ significantly (*p* < 0.05) (*n* = 8).

**Table 3:** Alpha diversity indices of rectal bacterial communities in piglets.

	Treatments			
Item	Group I	Group II	Group III	*p*-Value
Sobs	378.33 ± 8.08 ^a^	310.00 ± 26.87 ^b^	382.00 ± 27.22 ^a^	0.028
Coverage, %	99.88 ± 0.04	99.85 ± 0.03	99.90 ± 0.01	0.320
Shannon	4.08 ± 0.34 ^a^	3.39 ± 0.22 ^b^	3.83 ± 0.20 ^ab^	0.090
ACE	404.56 ± 16.93	355.59 ± 43.60	407.24 ± 24.15	0.167
Chao	407.27 ± 11.47	366.23 ± 42.23	410.28 ± 21.17	0.194

^a, b^ Means in the same row with different superscript letters differ significantly (*p* < 0.05) (*n* = 3).

**Table 4:** Different endogenous metabolites in feces of weaned piglets in group I, group II, and group III.

Metabolite Names	*m*/*z* ^1^	Retention Time ^2^ (min)	Group I vs. Group II			Group I vs. Group III			Group II vs. Group III			Main Functions
			log2(FC) ^3^	*p*-Value ^4^	Change ^5^	log2(FC)	*p*-Value	Change	log2(FC)	*p*-Value	Change	
L-Aspartic acid	134.04	93.54	−1.238	<0.001	↓	—	—	—	1.364	<0.001	↑	ABC transporters, biosynthesis of alkaloids derived from ornithine, lysine and nicotinic acid
(5-hydroxyindol-3-YL) acetic acid	192.06	421.81	−1.416	<0.001	↓	—	—	—	1.310	<0.001	↑	Phenylalanine metabolism
L-Phenylalanyl-L-Proline	263.14	375.67	—	—	—	1.119	0.001	↑	—	—	—	ABC transporters
2-Keto-3-Deoxy-D-Gluconic acid	177.04	100.44	—	—	—	1.466	0.003	↑	—	—	—	Glucose metabolism
Octadecanoic acid	282.94	402.91	—	—	—	−1.276	0.004	↓	−0.968	0.006	↓	Lipid metabolism
CMP	324.21	67.70	—	—	—	1.197	0.005	↑	—	—	—	Pyrimidine metabolism
Adipic acid	145.05	322.59	—	—	—	—	—	—	−0.622	0.003	↓	Lysine biosynthesis and degradation

^1^ *m*/*z*, mass-to-charge ratio; ^2^ RT, retention time; ^3^ FC, fold change; ^4^ *p*-value, a significant difference *p* value, FDR < 0.05; ^5^ “↑/↓” indicate the increase/decrease of the metabolite level; “—” means it does not exist (*n* = 6).

## Data Availability

The original contributions presented in the study are included in the article; further inquiries can be directed to the corresponding authors.
